# Acute Lyme disease IgG N-linked glycans contrast the canonical inflammatory signature

**DOI:** 10.3389/fimmu.2022.949118

**Published:** 2022-08-05

**Authors:** Benjamin Samuel Haslund-Gourley, Stéphane Grauzam, Anand S. Mehta, Brian Wigdahl, Mary Ann Comunale

**Affiliations:** ^1^ Department of Microbiology and Immunology, Drexel University College of Medicine, Philadelphia, PA, United States; ^2^ Institute for Molecular Medicine and Infectious Disease, Drexel University College of Medicine, Philadelphia, PA, United States; ^3^ GlycoPath, LLC Charleston, SC, United States; ^4^ Department of Cell and Molecular Pharmacology, Medical University of South Carolina (MUSC), Charleston, SC, United States

**Keywords:** Lyme disease, IgG N-glycans, glycosylation, immunoglobulin, diagnostics - clinical characteristics, biomarker

## Abstract

Lyme disease (LD) infection is caused by *Borrelia burgdorferi* sensu *lato* (Bb). Due to the limited presence of this pathogen in the bloodstream in humans, diagnosis of LD relies on seroconversion. Immunoglobulins produced in response to infection are differentially glycosylated to promote or inhibit downstream inflammatory responses by the immune system. Immunoglobulin G (IgG) N-glycan responses to LD have not been characterized. In this study, we analyzed IgG N-glycans from cohorts of healthy controls, acute LD patient serum, and serum collected after acute LD patients completed a 2- to 3-week course of antibiotics and convalesced for 70-90 days. Results indicate that during the acute phase of Bb infection, IgG shifts its glycosylation profile to include structures that are not associated with the classic proinflammatory IgG N-glycan signature. This unexpected result is in direct contrast to what is reported for other inflammatory diseases. Furthermore, IgG N-glycans detected during acute LD infection discriminated between control, acute, and treated cohorts with a sensitivity of 75-100% and specificity of 94.7-100%.

## Introduction

In 1975, a cluster of children and adults in the small community of Lyme, Connecticut experienced unusual arthritic symptoms. Today, Lyme disease (LD) is the most prevalent vector-borne disease in the US, with an estimated 476,000 annual cases (1). The causative agent, *Borrelia burgdorferi* sensu lato (Bb), is a spirochete transmitted from the *Ixodes* tick into the human host dermis during its blood meal (2). The spirochete leaves the blood and disseminates into multiple organ systems in as little as two weeks post-infection (3–5). Disseminated LD is more challenging to diagnose, and delayed treatment can lead to long-term disability or death (6, 7). The disease is endemic in the Northeastern US, and incidence rates continue to rise. The US’s annual treatment and diagnostic cost is over 4.8 billion USD (8).

Antibiotic treatment for LD in the acute phase is often curative (9–12). However, untreated patients and a subset of treated patients progress to disseminated disease (13, 14), Disseminated disease can result in facial nerve palsy (15), Lyme Carditis (16), Lyme Arthritis (17), Lyme Neuroborreliosis (18, 19), and long-term disability (20, 21). Persistent symptoms are reported by 10-20% of patients diagnosed and treated during the acute phase of LD. Persistent symptoms include joint pain, fatigue, and neurocognitive deficits (10, 22). This highlights the need for an accurate early diagnosis and the ability to track disease resolution.

Current diagnosis is complicated because testing relies on indirect methods. Direct PCR and blood culture methods often fail due to the spirochete’s limited presence in the bloodstream, low bacterial counts in circulation, slow replication cycle, the requirement for complex growth media, and specialized microscopy requirements (23, 24). Hence, indirect methods that rely on the patient’s serological response are the principal method of confirming an infection (25). These indirect methods of acute LD diagnostics based on ELISA and western immunoblot technologies suffer from low sensitivity and a high false-positive rate. Thus, while advances in LD detection research are being made (26–29), clinicians currently lack a sensitive method to diagnose early disease. Furthermore, clinical assays are unable to determine treatment efficacy, track disease resolution (30) or diagnose subsequent infections.

Serum protein glycosylation is often altered during inflammatory and autoimmune diseases. The glycosylation profile of immunoglobulins is dynamic and offers a novel immunologic insight into the host’s response (31–34). Glycosylation is the most abundant complex post-translational serum protein modification (35) and plays a significant role in protein structure and function *in vivo* (36, 37). IgG has a well-characterized N-glycosylation site on the constant fragment (Fc- Asn-297) region (38). This site contains complex biantennary glycans with varying degrees of galactose, bisecting N-acetyl-glucosamine, and sialic acid residues. Most notably, the glycans are highly core-fucosylated. IgG glycosylation is dynamic and the glycans present can affect the binding avidity to various Fcγ receptors, rate of complement activation, and release of cytokines (39–42). Previous studies have profiled IgG N-glycans in sera obtained from patients with inflammatory disease (**Table 1** **)**. Reports indicate a trend for IgG N-glycan reduction in terminal galactose and sialic acid content during inflammatory diseases and this glycan signature is linked to a pro-inflammatory phenotype (52–58).

**Table 1:** Altered IgG N-glycan Profiles Detected in Inflammatory Diseases.

Disease	IgG Fc N-Glycan Profile	Method of Detection	Source
Rheumatoid Arthritis (RA) and Osteoarthritis (OA)	Decreased Galactose Decreased Bisects Elevated Agalactose	Mass Spectrometry – MALDI-TOF	(32, 33)
Tuberculosis	Decreased Galactose	Radiolabels and sequential exoglycosidase digestion	(31, 43)
Infective endocarditis	Decreased Galactose	Lectin Analysis	(44)
Visceral leishmaniasis	Decreased Galactose Decreased Sialyation Decreased Bisects	Mass Spectrometry – MALDI-TOF	(45)
Hepatitis B: chronic infection	Decreased Galactose	Liquid Chromatography and Mass Spectrometry	(46)
Hepatitis C – anti-Gal IgG	Decreased Galactose Increased Fucose	Liquid Chromatography and Lectin Analysis	(47)
Liver Fibrosis	Increased Bisects Decreased Galactose	Mass Spectrometry – MALDI-Imaging	(48)
Inflammatory bowel disease & Crohn’s disease	Decreased Galactose Decreased Sialyation	Liquid Chromatography and Fluorescent Detection	(34)
Endometriosis	Decreased Sialylation & Galactose Increased Bisects	Liquid Chromatography and Fluorescent Detection	(49)
Systemic Lupus	Decreased Galactose Decreased Sialyation Decreased Fucose Increased Bisects	Liquid Chromatography and Fluorescent Detection	(50)
Moderate COVID-19	Decreased Galactose Increased Agalactose	Liquid Chromatography and Mass Spectrometry	(51)

Inflammatory diseases have serum IgG with N-glycan alterations that promote a pro-inflammatory signaling cascade due to a reduction in terminal galactose and sialic acid.

Scientists are beginning to understand how glycosylation reflects health status and influences protein structure and function. IgG glycosylation moieties are associated with specific functions. In general, lowered galactose and sialic acid residue content are reported in inflammatory states (**Table 1**), and conversely, increased galactose is associated with an anti-inflammatory state. Hence, to improve immunotherapy outcomes, pharmaceutical companies glycoengineer monoclonal antibodies with specific glycosylation features to treat cancers and chronic diseases. For example, the effectiveness of intravenous immunoglobulin (IVIG) therapy for autoimmune conditions including Gillian-Barre, Immune Thrombocytopenic Purpura, or Kawasaki disease patients is associated with the increased sialic acid content on the Fc N-glycans of the IVIG (59–61). In addition, therapeutic monoclonal antibodies are glycoengineered to contain specific a-fucosylated, agalactosylated N-glycans to promote superior half-life and treatment efficacy (62–65). IgG N-glycan modulation of the immune response during disease reveals another layer of physiologic crosstalk. Evidence indicates that the repertoire of N-glycans present on IgG produced in response to vaccines is dependent on many factors, including age, inflammatory state, and the type of adjuvant (58).

It is well accepted that N-glycans modulate the function of antibodies and are altered in disease states (66). We hypothesize that the IgG N-glycan profile of LD patients will reflect the immunological response to the acute LD infection. Thus, this first report of total IgG N-glycans associated with LD will provide insight into the inability of the host immune system to resolve Bb bacterial infection.

## Material and methods

### Patient samples

Serum samples (**Table 2**) were obtained from the Bay Area Lyme Disease Biobank and stored at -80°C. Serology was determined at Stony Brook University and the Bay Area Lyme Disease Biobank (67). Human subject research IRB requirements were met (IRB #1808006553). Acute LD patients presented with erythema migrans (EM) rash(es) and donated their blood at the time of clinical diagnosis. Acute LD patients were included in the cohort when their acute LD diagnosis was confirmed using either two-tiered serological studies or PCR identification. Convalescent draws were obtained after patients completed their prescribed course of 14-21 days of antibiotics and convalesced for 70-90 days without further symptoms. We refer to the convalescent cohort as “treated”. In the pilot study, serum was pooled into three patient cohorts. Seven healthy controls were pooled, 5 acute LD patients were pooled, and 3 treated LD patients were pooled. The resulting three samples were analyzed within the pilot study using HPLC and the GlycoTyper MALDI-TOF methods. In the subsequent confirmatory study using the GlycoTyper method, data was collected from each individual patient: healthy control (n=18), acute (n=18), and patient-matched treated (n=18). Further demographic details for the patient cohorts are presented in **Supplemental Table 1**.

**Table 2:** Patient Samples.

		Sample‘n’	Avg Age	% Female	% Hispanic Latino	% Wisconsin	% Long Island	Serum Collected after treatment?
**Pilot Set**	Control	n=7	46.4	42.9	42.9	0	100	Not Applicable
**Pilot Set**	Lyme disease	n=5 Acute n=3 Treated	51.4	40.0	40.0	0	100	Yes
**Confirmatory Set**	Control	n=18	51.3	40.9	22.7	18.2	81.8	Not Applicable
**Confirmatory Set**	Lyme disease	n=18 Acute n=18 Treated	53.1	40.0	20.0	20.0	80.0	Yes

Healthy Control and Lyme disease LD with matched Acute and Post-treatment Demographics of Bay Area Lyme Disease Biobank Serum Samples. Sample ID numbers are linked to demographic details including age, sex, ethnicity, and if the patient donated a post-treatment convalescent serum sample.

### HPLC IgG N-glycan analysis

IgG was purified from 5μL of pooled serum using Protein A/G UltraLink Resin (Thermo Scientific, MA) according to the manufacturer’s directions. IgG Heavy Chains (Fc region) were isolated using 1D gel electrophoresis, stained with Coomassie stain and the 50kDa band was excised. Following gel de-staining, the glycans were enzymatically removed and fluorescently labeled following standard in-gel PNGase F and labeling protocols as previously described (68, 69). The labeled N-glycans were combined with 100% Acetonitrile (30:70) in an HPLC-compatible vial. Fluorescently labeled glycans were subsequently analyzed by high-performance liquid chromatography (HPLC) by using a TSK-Gel Amide 80 column (Tosoh Bioscience LLC). The mobile phase consisted of solvent A (50 mmol/L ammonium formate, pH 4.4) and solvent B (acetonitrile). The gradient used was as follows: a linear gradient from 20% to 58% solvent A at 0.4 mL/min for 152 min followed by a linear gradient from 58% to 100% solvent A for the next 3 minutes. The flow rate was increased to 1.0 mL/min, and the column was washed in 100% solvent A for 5 minutes. Following the wash step, the column was equilibrated in 20% solvent A for 22 minutes in preparation for the next sample run. HPLC analysis was performed using the Waters Alliance HPLC System, complemented with a Waters fluorescence detector, and quantified by the Millennium Chromatography Manager (Waters Corporation). Glycan structures were identified by calculating the glucose unit and GlycoStore database as previously described (70). The HPLC method overview is provided in **Supplemental Figure 1A**.

### Removal of sialic acids for HPLC and MALDI comparison

Due to the inherent challenges of detecting glycans containing sialic acid using mass spectrometry, HPLC data was collected using samples that were desialylated to allow a direct comparison with the MALDI-FT-ICR method. Beginning with 13μL of the labeled and chromatographically cleaned 2-AB glycans, 4μL 5X pH 6.0 Enzyme Buffer was added before pipette mixing 3μL of Sialidase A (ProZyme (now AdvanceBio), OH). The 20μL final volume was incubated at 32°C for 12 hours. Next, the 20μL solution was added to a 10K MWCO concentrator column (Corning, NY) and centrifuged at 12,000 rpm for 10 minutes. The flow-through was collected and combined with an additional 25μL of dH2O before being added back to the 10K MWCO column and centrifuged at 12,000 rpm. This process of collecting the flow-through and combining it with additions of 25μL dH2O was repeated to serially enrich the labeled N-glycans while filtering out the sialidase enzyme. The sample was subsequently dried down *via* SpeedVac holding a vacuum at -28in Hg without added heat and re-suspended in 30μL dH2O. Sialidase-treated samples were then analyzed as outlined for non-sialidase-treated sample in HPLC.

### MALDI-FT-ICR mass spectrometry N-glycan analysis

SolarisX Legacy 7T FT-ICR mass spectrometer equipped with a Matrix-Assisted Laser Desorption/Ionization (MALDI) (Bruker) analysis of total IgG N-glycans is detailed in the literature (48, 71–73). In brief, 1μL serum diluted in 99μL 1X PBS was incubated with 0.2mg/ml spotted anti-IgG capture antibodies (Bethyl Laboratories Inc., Tx, Cat. Number A80-104) treated with Sialidase A (produced in-house by MUSC), sprayed with PNGase F (produced by N-Zyme, PA) to release N-glycans from captured targets, coated with a matrix, and analyzed for glycan abundance at specific m/z peaks by MALDI-FT-ICR MS using SCiLS Lab software 2022a (Bruker). A capture antibody treated with PBS served as a blank to subtract the N-glycans released from the capture antibody from the final analysis. A MALDI-FT-ICR (referred to as MALDI) method overview is provided in **Supplemental Figure 1B** and is also referred to as the “GlycoTyper” method.

### Statistical analysis

One-way ANOVA analysis with *post-hoc* Tukey’s multiple comparisons test was employed to examine the triplicate datasets for statistically significant differences between cohorts. P<0.05 was considered statistically significant and figures are denoted as having *p<0.05, **p<0.01, ***p<0.001. Figures and statistical analysis were completed using GraphPad Prism 8. Receiver Operating Characteristic (ROC) curves analyzed individual N-glycan abundance levels for diagnostic utility. Results are described using the Oxford nomenclature (74).

## Results

We demonstrate that total serum IgG N-glycosylation of acutely infected Lyme disease patients contrasts with the typical pro-inflammatory signature often found on other inflammatory diseases. First, a proof-of-concept study was performed using pooled serum to identify the IgG N-glycan signature of healthy control, acute LD, and patient-matched antibiotic-treated LD serum using high-pressure liquid chromatography separation (HPLC) and detection of fluorescently labeled glycans (75). The samples were subsequently analyzed using the recently developed GlycoTyper MALDI method (73) which pairs a specific total-IgG capture antibody with subsequent MALDI-FT-ICR imaging (MALDI). The trends in the glycan signatures were reproducible between both platforms, and thus we proceeded to analyze a larger confirmatory set of individual serum samples using the GlycoTyper platform.

### Acute LD IgG N-glycans gain terminal galactose and sialic acid

**Figure 1:**
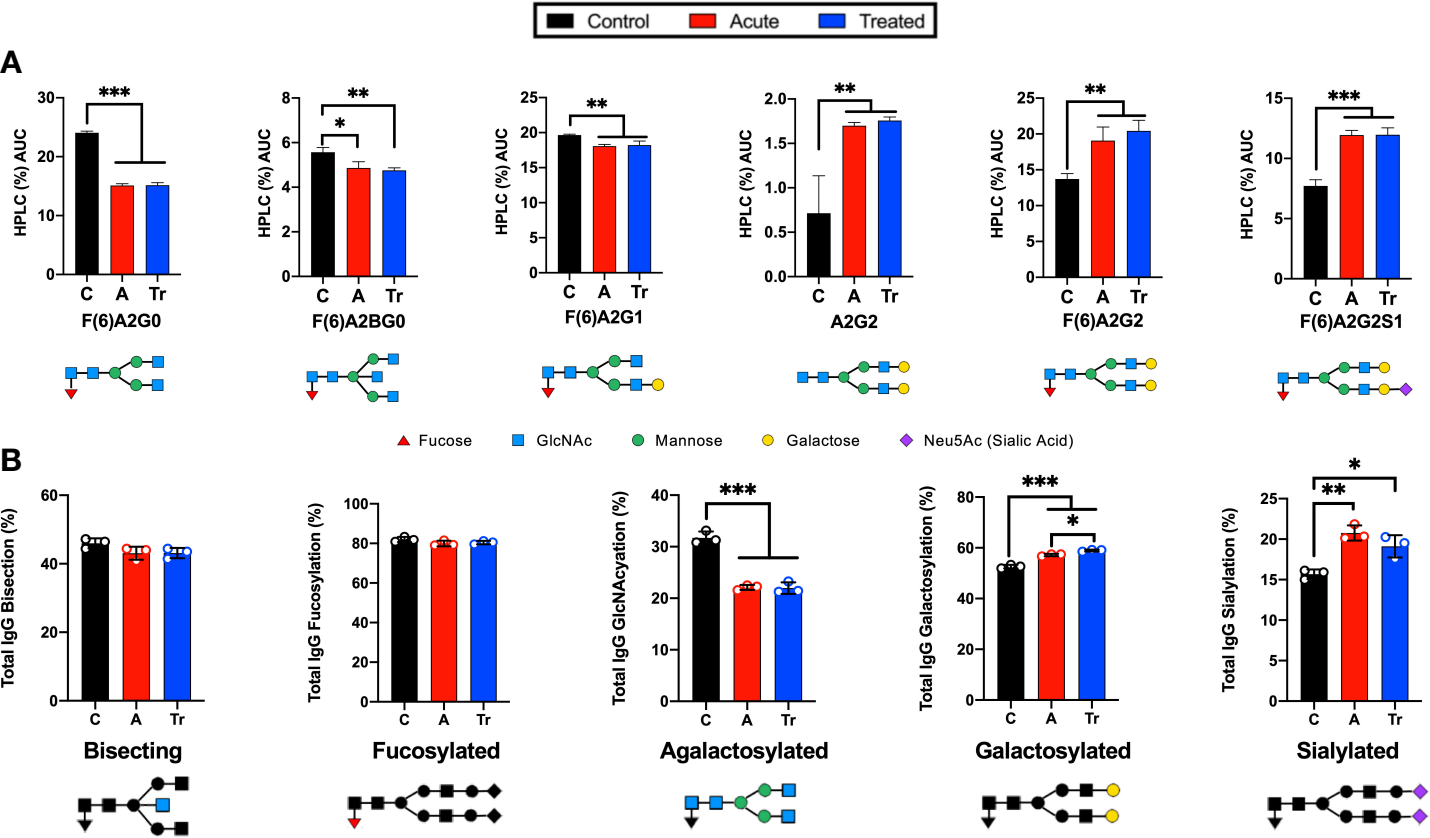
Analysis of IgG reveals significant changes in N-glycan structure distribution during acute and treated LD. **(A)** Significant alterations of IgG N-glycans reported by HPLC analysis from pooled serum from control (C), acute (A), or treated (Tr) patients reported as the percent area under the curve (AUC) for each peak determined from the average of triplicate samples. N-glycan structures are displayed below. Control represents pooled healthy age-matched serum n=7, Acute represents pooled serum from two-tiered diagnosed Lyme disease patients n=5, Treated represents patients donating serum a second time 70-90 days after completion of the reportedly curative round of antibiotic treatment (14-21d doxycycline) for Lyme disease n=3. **(B)** Labeled N-glycan classes: Bisecting, Fucosylated, Agalactosylated, Galactosylated, Sialylated detected using HPLC analysis of IgG N-glycans from 3 replicates +/- S.D. Analysis was completed using One-Way ANOVA with *post-hoc* Tukey’s multiple comparisons, *p<0.05, **p<0.01, ***p<0.001.

HPLC analysis of the glycan signature revealed several statistically significant differences between control and acutely infected patients (**Figure 1**). Individual glycans were quantitated as a percent of the total glycan profile and compared across cohorts using one-way ANOVA (**Figure 1A**). Two agalactosylated glycan species, F(6)A2G0 and F(6)A2BG0, decreased in the acute and treated pooled cohorts when compared to controls. There was also an observed decrease in the mono-galactosylated F(6)A2G1 glycan. In addition, we observed significant increases in three glycan species. N-glycans containing terminal di-galactose, with and without core fucose increased significantly (A2G2, F(6)A2G2), as did the core fucosylated mono-sialylated glycan (F(6)A2G2S1). Next, the abundance of IgG N-glycans with specific characteristics were summed to analyze the glycans by class. IgG N-glycans containing bisecting GlcNAc, core-fucose, no galactose, a mono- or digalactose, or a mono- or di-sialylated were respectively summed to present N-glycan class data (**Figure 1B**). The decrease in the smaller agalactosylated class and an increase in the terminally galactosylated and sialylated glycans was observed. However, bisecting and core-fucosylated N-glycan classes did not significantly vary across cohorts. The only statistical difference observed between the acute and treated patient cohorts was a continued increase in the presence of terminal galactose.

### MALDI-FT-ICR and HPLC detect similar trends of IgG N-glycans in LD

**Figure 2:**
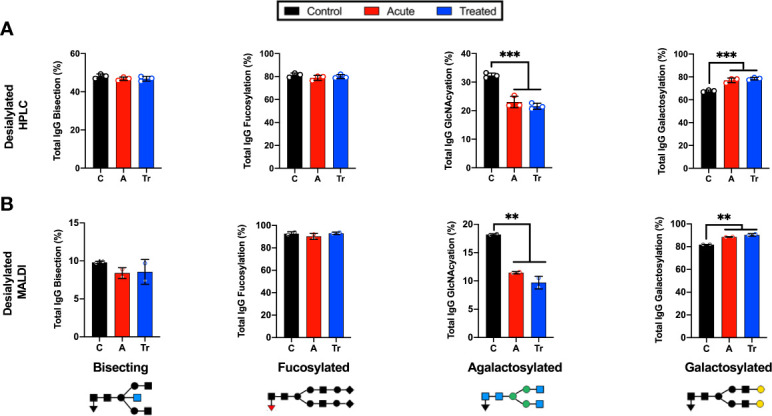
Grouping IgG N-glycans by class reveals MALDI and HPLC detect similar trends during LD **(A)** Labeled N-glycan classes: Bisecting, Fucosylated, Agalactosylated, Galactosylated, Sialylated detected using HPLC analysis of desialylated IgG N-glycans grouped by class averaged from 3 replicates of pooled cohorts described in **Figure 1**. **(B)** MALDI analysis of desialylated IgG N-glycan classes averaged from 2 replicates of pooled cohorts described in **Figure 1**. Analysis was completed using One-Way ANOVA with *post-hoc* Tukey’s multiple comparisons, **p<0.01, ***p<0.001.

IgG N-glycans were desialylated and the HPLC analysis was compared to the MALDI-FT-ICR method. The IgG N-glycans were grouped by terminal sugar moiety into 4 glycan classes. These groups showed the same trends using both platforms (**Figure 2**). Significant reductions in agalactosylated N-glycans and significant increases in terminal galactose were identified in the HPLC (**Figure 2A**) and MALDI glycan analysis platforms (**Figure 2B**). Both methods indicate there is no significant difference in the abundance of bisecting or core-fucosylated N-glycans when comparing controls, acute and treated patient cohorts. The reproducible shifts in IgG N-glycan abundance within the desialylated glycan classes promoted the use of a larger confirmatory set of LD serum to be analyzed in a high-throughput manner using the MALDI method.

### MALDI analysis identifies IgG N-glycosylation signatures of patients with LD

**Figure 3:**
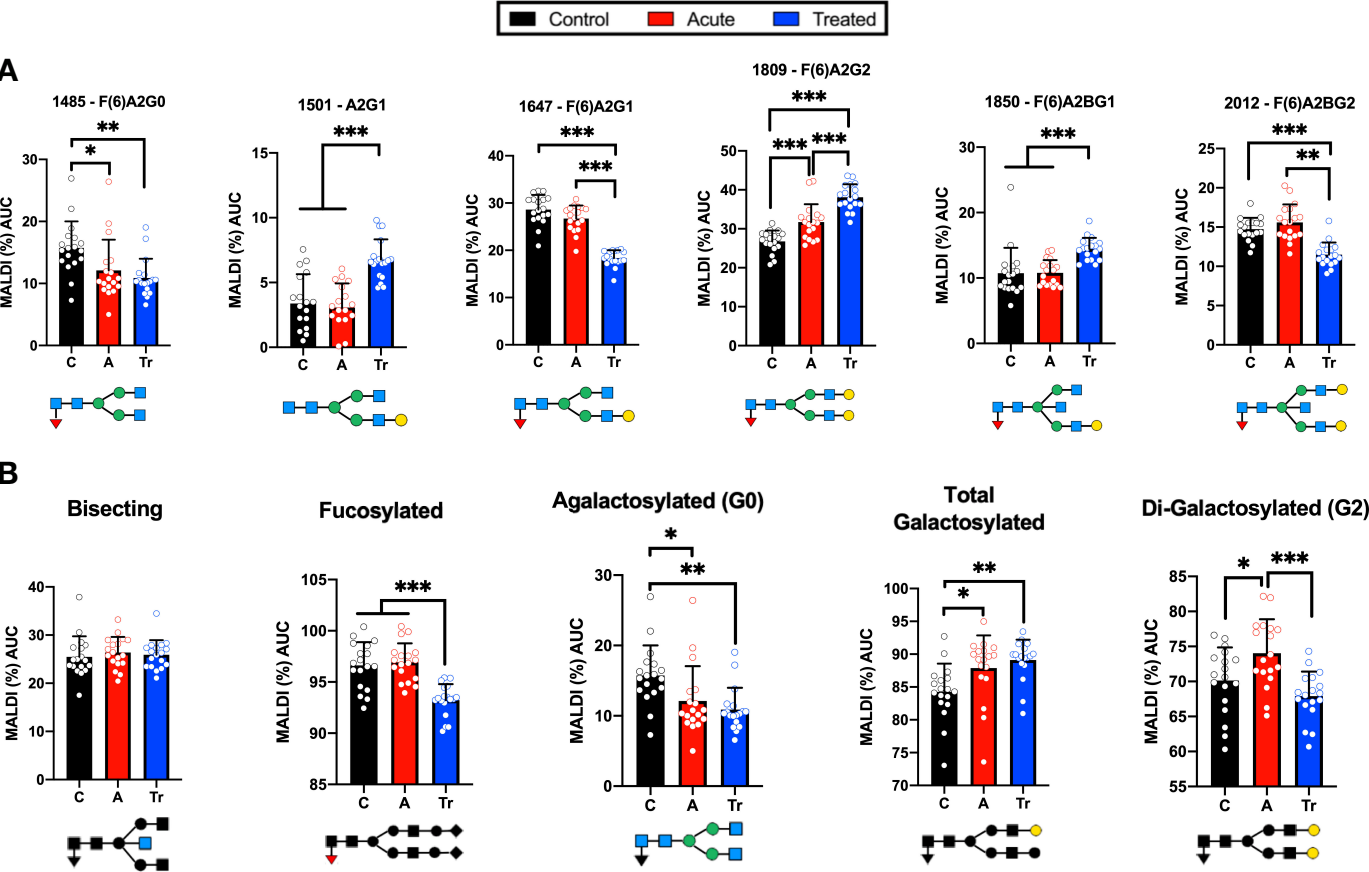
Confirmatory set of LD patient serum assayed using the high-throughput MALDI method **(A)** IgG N-glycans detected by MALDI analysis of Ab-captured IgG from n=18 cohorts individually run in triplicate +/- S.D. and reported as the percent of the total identified N-glycan m/z peak intensity. N-glycan structures displayed below **(B)** MALDI analysis of desialylated IgG N-glycans grouped by class from n=18 samples per cohort averaged in triplicate +/- S.D. Labeled N-glycan class structures: Bisecting, Fucosylated, Agalactosylated, Galactosylated, and Di-Galactosylated are presented below the respective graph. Analysis was completed using One-Way ANOVA with *post-hoc* Tukey’s multiple comparisons, *p<0.05, **p<0.01, ***p<0.001.

IgG N-glycans from a confirmatory set of healthy control, acute LD, and acute re-drawn serum post-antibiotic treatment patient sera were examined. MALDI analysis of the desialylated IgG N-glycans identified statistically significant shifts of N-glycan species (**Figure 3**). Several significant differences were detected when comparing control and acute LD. Acute LD patients exhibit a significant decrease in the core fucosylated agalactosylated N-glycan F(6)A2G0 when compared to controls (**Figure 3A**). Conversely, there was a statistically significant increase in the core fucosylated di-galactosylated N-glycan F(6)A2G2. IgG N-glycans terminating in total galactose or digalactose significantly increased during acute LD compared to healthy controls (**Figure 3B**). There was no significant difference in the total bisecting or core-fucosylated N-glycans when comparing Acute to healthy control IgG.

We observed a continued perturbation of IgG glycosylation in the post-treatment cohort that does not return towards healthy control baselines for many detected N-glycans. Treated LD patients maintained a significant decrease in the agalactosylated F(6)A2G0 observed in acute LD (**Figure 3A**). An additional significant drop was seen in two glycan structures, F(6)A2G1 and F(6)A2BG2, when compared with control and acute. Several glycans showed significant increases. Increases that were limited to the treated cohort include the A2G1 and F(6)A2BG1 structures. The F(6)A2G2 continued to increase above the already elevated acute LD cohort level. Grouping the treated timepoint IgG N-glycans revealed a drop in total core-fucose as well as a decrease in the digalactosylated grouped N-glycans (**Figure 3B**). There was no difference observed for the total bisecting N-glycans between acute and treated timepoints.

These findings align with previous assays from the pooled pilot study experiments. Comparisons of IgG N-glycosylation dependence on age, sex, location of collection, or ethnicity were assessed. There was no statistically significant difference within or between cohorts when applying these metrics (data not shown) with one exception. Healthy control males had 2.3% higher F(6)A2G0 N-glycan profiles compared to their healthy control female counterparts. While IgG N-glycan signatures change during ageing and between sexes, the age- and sex-matched cohorts permitted comparisons across and between the cohorts (76).

### IgG N-glycans discriminate between healthy control and LD timepoints

**Figure 4:**
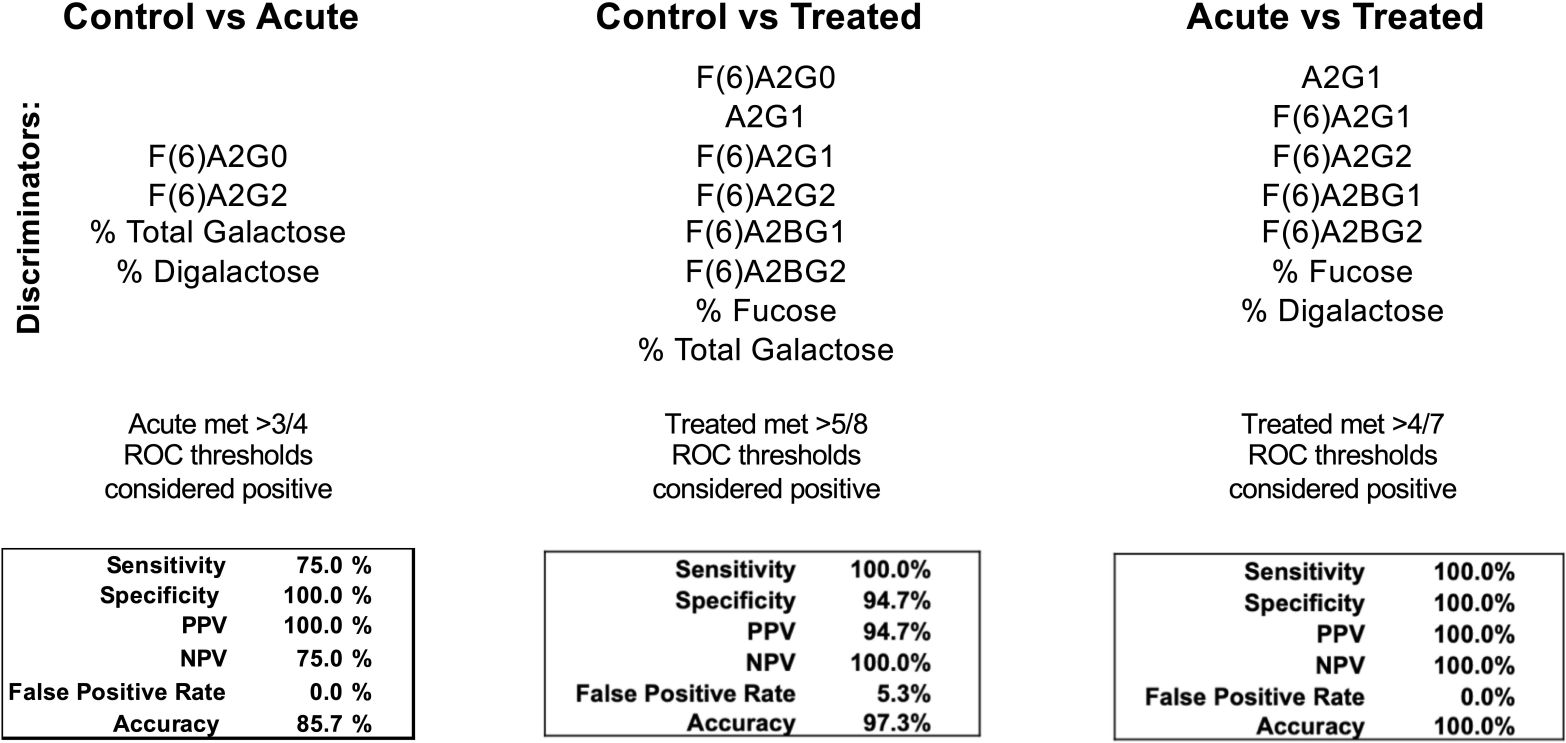
Confusion matrices from combined discriminatory IgG N-glycan ROC thresholds. N-glycans with significant discrimination between cohorts determined using ROC analysis are listed above. The listed N-glycan discriminators were combined for each cohort comparison (Control vs Acute LD, Control vs Treated LD, Treated vs Acute LD) and the resulting confusion matrices are reported.

Seroconversion is often employed as a LD diagnostic, yet there is great need for a more sensitive, early indicator of disease. Moreover, serological assays for LD cannot differentiate between a patient with acute LD compared to a patient that has recently convalesced from LD. **Figure 4** reports the efficacy of selected IgG N-glycans to discriminate between healthy and LD patient cohorts. Thresholds for listed N-glycan classes were determined using receiver operating characteristic (ROC) curve. The performance of the discrimination was reported in a confusion matrix (**Supplemental Figures 2**-**4**). Healthy controls are differentiated from acute serum samples using four N-glycan classes: F(6)A2G0, F(6)A2G2, percent total terminal galactose, and percent terminal di-galactose; resulting in 75% sensitivity, 100% specificity, and 85.7% accuracy. Healthy controls are differentiated from treated LD patient serum using the N-glycan classes: F(6)A2G0, A2G1, F(6)A2G1, F(6)A2G2, F(6)A2BG2, percent total fucose, and percent total terminal galactose; resulting in 100% sensitivity, 94.7% specificity, and 97.3% accuracy. Lastly, acute LD serum is differentiated from the treated serum using the N-glycan classes: A2G1, F(6)A2G1, F(6)A2G2, F(6)A2BG1, F(6)A2BG2, total percent fucose, and percent G2 galactosylation; resulting in 100% sensitivity, specificity, and accuracy.

### Lyme disease subverts IgG response – working hypothesis

**Figure 5:**
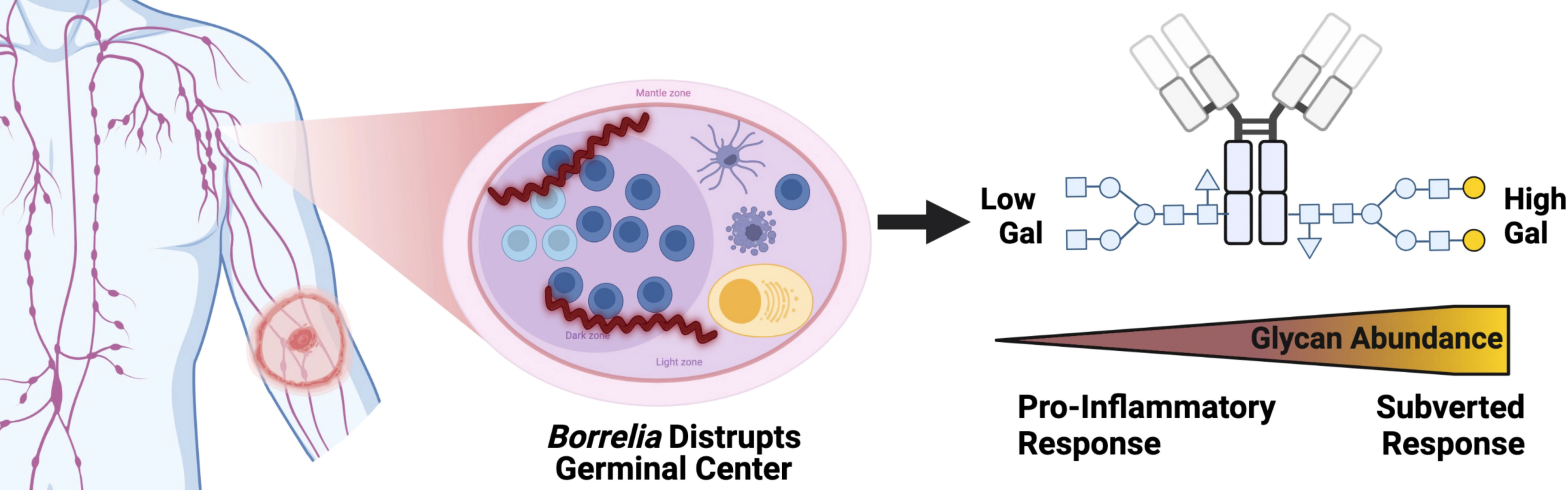
IgG N-glycans Respond to acute Lyme disease infection with increased galactose, impairing bound IgG to signal for inflammation, unlike other pro-inflammatory disease IgG signatures. Left to right, a tick infected with *Borrelia* transmits the infection to the human host leading to LD. The Bb spirochete disrupts the architecture of the germinal center within the lymph node. IgG molecules produced. Created with BioRender.com.

Results presented in **Figure 5** show the increased terminal galactose observed on IgG N-glycans detected during LD within the context of the humoral immune response. IgG N-glycans detected in acute LD patients contain significantly higher levels of galactose and conversely lower agalactosylated structures. Most IgG N-glycans responding to inflammatory diseases present increased agalactosylated N-glycans to promote downstream pro-inflammatory immune responses. Thus, the LD IgG N-glycans may be induced through the Borrelia *burgdorferi’s* disruption of the germinal center and antibody response.

## Discussion

This is the first report of total human IgG N-glycan profiles during early and treated LD. Our initial studies detected an unanticipated shift in IgG N-glycan profile which was confirmed and expanded upon using a larger set of samples. During many inflammatory states (**Table 1**), IgG N-glycan profiles respond with reduced terminal sialic acids and galactose and increased GlcNAc sugar exposure (39). This agalactosylated IgG phenotype has been demonstrated to promote pro-inflammatory responses and may aid in clearing the infection (52–58, 77, 78). In the case of LD, total IgG N-glycan profiles had the opposite trend. LD increases the galactose and sialic acid content of IgG N-glycans while further decreasing the terminal GlcNAc exposure (**Figures 1** **–** **4**). The result of such a shift requires further investigation, but in theory could contribute to ineffective host responses to LD infection by reducing humoral immune activation (77, 78). The ability to detect these changes within total IgG suggests that LD induces a large shift in the IgG N-glycan composition. Thus, this dysregulated immune response may at least in part explain why human IgG produced during an initial LD infection is not effective to clear the current infection or protect from a future re-infection with LD.

The pilot study served to compare the well-established HPLC IgG N-glycan analysis method (79) to the novel MALDI analysis method (73) using pooled cohorts of control, acute LD, and treated LD serum. Due to MALDI’s limited detection of unmodified sialic acids, samples were treated with a sialidase (72). The results presented in **Figure 2** demonstrate that both methods detect comparable individual identified N-glycans with similar trends of terminal galactose exposure induced during LD and maintained during the post-antibiotic treatment time points. To confirm the pooled pilot study findings, three cohorts of 18 individual samples were analyzed in triplicate using the MALDI method. The GlycoTyper method was selected to analyze the confirmatory cohort due to its significantly higher throughput compared to HPLC. We found that the demographics of the age- and sex-matched patient cohorts did not lead to inherent differences in IgG N-glycosylation.

While the MALDI results confirmed the original trends observed in the pooled pilot study, a higher number of samples analyzed led to further trends emerging (**Figures 3A, B** **)**. Agalactosylated structures are reduced while total N-glycans terminating with a galactose moiety continued to increase within the acute and treated cohorts as indicated within the pooled pilot experiments. N-glycans containing a core-fucose are markedly lowered in the antibiotic-treated cohort which suggests an increase in Antibody-Dependent Cellular Cytotoxicity (ADCC) abilities of post-treatment IgG (80). The increased level of digalactosylated (G2) N-glycans on IgG at the acute stage and subsequent return to healthy control levels is a potential biomarker reflecting the host’s response to successful antibiotic treatment. Following antibiotic therapy for Tuberculosis (TB), IgG N-glycan digalactose content returned to healthy control levels (43). Grace et al. found G2 N-glycans decreased during acute TB and subsequently increased after effective antibiotic treatment. In the case of LD, IgG N-glycans increase G2 content during infection and return to healthy control levels after effective antibiotic treatment (**Figure 3B** **)**. This once more converse trend observed in LD suggests a subverted immune response during LD compared to TB infection. The implication of the IgG N-glycan response to LD is portrayed in **Figure 5**.

LD patients are discriminated from healthy controls with a high degree of sensitivity (75-100%) and specificity (94.7-100%) using total IgG N-glycan measurements (**Figure 4**). Blinded analysis of acute LD, post-treatment LD, and mimic diseases using these methods should be completed to validate these findings. Future LD tests incorporating total IgG N-glycan analysis could increase acute LD diagnostic sensitivity and track subsequent antibiotic treatment responses.

Mechanisms controlling the dynamic B-cell glycosyltransferase expression operating on IgG N-glycans during LD require examination. B-cell glycosyltransferase expression is known to respond to the cytokine micro-environment (81). Recent studies detected IgG N-glycosylation profiles are impacted by the type of adjuvant present during vaccination (82). Additionally, during the onset of autoimmune disease, T_H_17 cells signal newly differentiated B cells with IL-22 and IL-23 to regulate glycosyltransferase expression, resulting in a pro-inflammatory agalactosylated, non-fucosylated IgG N-glycan (83). The increase in terminal galactose of IgG N-glycans during acute LD may be attributed to an upregulation of naïve B-cell beta-1,4-galactosyltransferase expression (66, 84–86). Future studies should determine if LD induces a specific cytokine signaling pathway to affect the glycosyltransferase expression of plasma cells or B cells during LD infection.

Lyme disease has been demonstrated to destroy germinal centers of lymph nodes during early infection (77, 78, 87, 88). These germinal centers are a vital structure that produces long-lived immunoglobulin responses through T-cell-dependent interactions (89). The destruction of the germinal center may explain why patients are liable to become re-infected with LD after treatment. Additionally, Bb has been demonstrated to gain entry inside human endothelial cells (90), alter innate immune responses after phagocytosis (91), suppress lymphocytes growth rate (92), and down-regulate major-histocompatibility complexes expression on Langerhans cells (93). Any one of these effects could be responsible for altering the IgG N-glycan profile during LD. For example, antigens presented in a T-cell *independent* manner promote increased sialyation on the Fc portion of IgG leading to an immunotolerant, less pro-inflammatory IgG N-glycan repertoire (94). Interestingly, murine studies have demonstrated a reduction in the general humoral response after LD infection as indicated by low titers of anti-viral antibodies produced post-vaccination and Bb-impaired helper T-cell mediated affinity maturation (87, 95). Lastly, because the LD cohort completed 2-3 week of oral doxycycline therapy, the serum N-glycome or immune response could have been altered in part due to changes in gut flora (96, 97).

## Conclusion

Using the GlycoTyper MALDI-FT-ICR imaging approach, we detected an unexpected IgG N-glycan signature in humans during LD. We have demonstrated the IgG N-glycans produced during a Lyme disease infection lack the classic highly agalactosylated signature associated with most inflammatory diseases. Instead, LD induces IgG N-glycans containing larger, terminally galactosylated sugar moieties. Moreover, many IgG N-glycans detected at the acute LD timepoint remain altered from healthy control levels at the post-antibiotic treatment timepoint. Of note, the digalactose content of IgG N-glycans in the treated time point was comparable to the healthy baseline levels after antibiotic treatment. Furthermore, we detected a significant decrease in total core-fucosylation at the treated timepoint suggesting a possible increase in ADCC which may aid in clearing Bb from the host. IgG N-glycans offer numerous biomarkers, reflect acute disease state, response to treatment, and may improve the sensitivity of the acute diagnosis of LD above the current two-tiered testing protocol. This first examination of IgG N-glycan signatures associated with LD requires future study.

## Data availability statement

The raw data supporting the conclusions of this article will be made available by the authors, without undue reservation.

## Ethics statement

The studies involving human participants was reviewed and approved by the University of Drexel College of Medicine Review Board. This study involves secondary research using de-identified data and biospecimens not collected specifically for this study. The IRB 1808006553 was assigned an exempt status.

## Author contributions

BH-G and MC were involved in conceptualization of assay, assay design, data analysis, and manuscript writing. BH-G was responsible for statistical analysis. AM and SG were involved in assay design and data analysis. BW was involved in editing the manuscript. All authors contributed to the article and approved the submitted version.

## Funding

This research was funded through the Mary K. Dewitt Pettit MD, Fellowship Fund (Award number: 282975) and through the Institute for Molecular Medicine and Infectious Disease at Drexel University College of Medicine.

## Acknowledgments

We thank Mengjun Wang and Dr. Laura Steel for their support. We also thank Dr. Elizabeth Horn for her partnership through the Bay Area Lyme Disease Biobank.

## Conflict of interest

Author AM is a founding partner in GlycoPath, Inc. and holds patents for the GlycoTyper methodology.

The remaining authors declare that the research was conducted in the absence of any commercial or financial relationships that could be construed as a potential conflict of interest.

## Publisher’s note

All claims expressed in this article are solely those of the authors and do not necessarily represent those of their affiliated organizations, or those of the publisher, the editors and the reviewers. Any product that may be evaluated in this article, or claim that may be made by its manufacturer, is not guaranteed or endorsed by the publisher.

## References

[1] Kugeler KJ Schwartz AM Delorey MJ Mead PS Hinckley AF. Estimating the frequency of Lyme disease diagnoses, united states, 2010–2018. Emerg Infect Dis (2021) 27(2):616–9. doi: 10.3201/eid2702.202731 PMC7853543 33496229

[2] Hugli D Moret J Rais O Moosmann Y Erard P Malinverni R. Tick bites in a Lyme borreliosis highly endemic area in Switzerland. Int J Med Microbiol (2009) 299(2):155–60. doi: 10.1016/j.ijmm.2008.06.001 18722157

[3] Dhruv C. Spatiotemporal evolution of erythema migrans, the hallmark rash of Lyme disease. Biophys J (2014) 106(3):763–8. doi: 10.1016/j.bpj.2013.12.017 PMC3944903 24507617

[4] Donta ST States LJ Adams WA Bankhead T Baumgarth N Embers ME. Report of the pathogenesis and pathophysiology of Lyme disease subcommittee of the HHS tick borne disease working group. Front Med (Lausanne) (2021) 8:643235. doi: 10.3389/fmed.2021.643235 34164410 PMC8215209

[5] Bender PD Ilgen JS. Early disseminated Lyme disease. BMJ Case Rep (2018) 2018:bcr-2017-223889. doi: 10.1136/bcr-2017-223889 PMC5935170 29724873

[6] Mac S Da Silva SR Sander B. The economic burden of Lyme disease and the cost-effectiveness of Lyme disease interventions: A scoping review. PloS One (2019) 14(1):e0210280. doi: 10.1371/journal.pone.0210280 30608986 PMC6319811

[7] Zhang X Meltzer MI Peña CA Hopkins AB Wroth L Fix AD. Economic impact of Lyme disease. Emerg Infect Dis (2006) 12(4):653–60. doi: 10.3201/eid1204.050602 PMC3294685 16704815

[8] Davidsson M. The financial implications of a well-hidden and ignored chronic Lyme disease pandemic. Healthcare (Basel) (2018) 6(1):16. doi: 10.3390/healthcare6010016 PMC5872223 29438352

[9] Maloney EL. Evidence-based, patient-centered treatment of erythema migrans in the united states. Antibiotics (2021) 10(7):754. doi: 10.3390/antibiotics10070754 34206379 PMC8300839

[10] Cameron DJ Johnson LB Maloney EL. Evidence assessments and guideline recommendations in Lyme disease: the clinical management of known tick bites, erythema migrans rashes and persistent disease. Expert Rev Anti Infect Ther (2014) 12(9):1103–35. doi: 10.1586/14787210.2014.940900 PMC4196523 25077519

[11] Roome A Spathis R Hill L Darcy J Garruto R. Lyme Disease transmission risk: Seasonal variation in the built environment. Healthcare (2018) 6(3):84. doi: 10.3390/healthcare6030084 PMC6163686 30029458

[12] Bhatia B Hillman C Carracoi V Cheff BN Tilly K Rosa PA. Infection history of the blood-meal host dictates pathogenic potential of the Lyme disease spirochete within the feeding tick vector. PloS Pathog (2018) 14(4):e1006959. doi: 10.1371/journal.ppat.1006959 29621350 PMC5886588

[13] Norman MU Moriarty TJ Dresser AR Millen B Kubes P Chaconas G. Molecular mechanisms involved in vascular interactions of the Lyme disease pathogen in a living host. PloS Pathog (2008) 4(10):e1000169. doi: 10.1371/journal.ppat.1000169 18833295 PMC2542414

[14] Casselli T Divan A Vomhof-Dekrey EE Tourand Y Pecoraro HL Brissette CA. A murine model of Lyme disease demonstrates that borrelia burgdorferi colonizes the dura mater and induces inflammation in the central nervous system. PloS Pathog (2021) 17(2):e1009256. doi: 10.1371/journal.ppat.1009256 33524035 PMC7877756

[15] Halperin JJ. Nervous system Lyme disease: Diagnosis and treatment. Curr Treat Options Neurol (2013) 15(4):454–64. doi: 10.1007/s11940-013-0240-y 23666548

[16] Lelovas P Dontas I Bassiakou E Xanthos T. Cardiac implications of Lyme disease, diagnosis and therapeutic approach. Int J Cardiol (2008) 129(1):15–21. doi: 10.1016/j.ijcard.2008.01.044 18508142

[17] Arvikar SL Steere AC. Diagnosis and treatment of Lyme arthritis. Infect Dis Clin North Am (2015) 29(2):269–80. doi: 10.1016/j.idc.2015.02.004 PMC4443866 25999223

[18] Berglund J Eitrem R Ornstein K Lindberg A Ringnér Å Elmrud H. An epidemiologic study of Lyme disease in southern Sweden. N Engl J Med (1995) 333(20):1319–24. doi: 10.1056/NEJM199511163332004 7566023

[19] Cardenas-de la Garza JA de la Cruz-Valadez E Ocampo-Candiani J Welsh O. Clinical spectrum of Lyme disease. Eur J Clin Microbiol Infect Dis (2019) 38(2):201–8. doi: 10.1007/s10096-018-3417-1 30456435

[20] Bratton RL Whiteside JW Hovan MJ Engle RL Edwards FD. Diagnosis and treatment of Lyme disease. Mayo Clin Proc (2008) 83(5):566–71. doi: 10.1016/S0025-6196(11)60731-3 18452688

[21] Shor S Green C Szantyr B Phillips S Liegner K Burrascano J. Chronic Lyme disease: An evidence-based definition by the ILADS working group. Antibiotics (2019) 8(4):269. doi: 10.3390/antibiotics8040269 PMC6963229 31888310

[22] Maloney EL. Controversies in persistent (Chronic) Lyme disease. J Infus Nurs (2016) 39(6):369–75. doi: 10.1097/NAN.0000000000000195 PMC5102277 27755213

[23] Marques AR. Laboratory diagnosis of Lyme disease. Infect Dis Clin North Am (2015) 29(2):295–307. doi: 10.1016/j.idc.2015.02.005 25999225 PMC4441761

[24] Hyde JA. Borrelia burgdorferi keeps moving and carries on: A review of borrelial dissemination and invasion. Front Immunol (2017) 8:114. doi: 10.3389/fimmu.2017.00114 28270812 PMC5318424

[25] Lantos PM Auwaerter PG Nelson CA. Lyme Disease serology. JAMA (2016) 315(16):1780. doi: 10.1001/jama.2016.4882 27115380 PMC5491346

[26] Tokarz R Mishra N Tagliafierro T Sameroff S Caciula A Chauhan L. A multiplex serologic platform for diagnosis of tick-borne diseases. Sci Rep (2018) 8(1):3158. doi: 10.1038/s41598-018-21349-2 29453420 PMC5816631

[27] Chou E Lasek-Nesselquist E Taubner B Pilar A Guignon E Page W. A fluorescent plasmonic biochip assay for multiplex screening of diagnostic serum antibody targets in human Lyme disease. PloS One (2020) 15(2):e0228772. doi: 10.1371/journal.pone.0228772 32040491 PMC7010292

[28] Schutzer SE Body BA Boyle J Branson BM Dattwyler RJ Fikrig E. Direct diagnostic tests for Lyme disease. Clin Infect Dis (2019) 68(6):1052–7. doi: 10.1093/cid/ciy614 PMC6399434 30307486

[29] Moore A Nelson C Molins C Mead P Schriefer M. Current guidelines, common clinical pitfalls, and future directions for laboratory diagnosis of Lyme disease, united states. Emerg Infect Dis (2016) 22(7):1169–77. doi: 10.3201/2207.151694 PMC4918152 27314832

[30] Bobe JR Jutras BL Horn EJ Embers ME Bailey A Moritz RL. Recent progress in Lyme disease and remaining challenges. Front Med (Lausanne) (2021) 8:666554. doi: 10.3389/fmed.2021.666554 34485323 PMC8416313

[31] Parekh R Isenberg D Rook G Roitt I Dwek R Rademacher T. A comparative analysis of disease-associated changes in the galactosylation of serum IgG. J Autoimmun (1989) 2(2):101–14. doi: 10.1016/0896-8411(89)90148-0 2504180

[32] Parekh RB Dwek RA Sutton BJ Fernandes DL Leung A Stanworth D. Association of rheumatoid arthritis and primary osteoarthritis with changes in the glycosylation pattern of total serum IgG. Nature (1985) 316(6027):452–7. doi: 10.1038/316452a0 3927174

[33] Sun D Hu F Gao H Song Z Xie W Wang P. Distribution of abnormal IgG glycosylation patterns from rheumatoid arthritis and osteoarthritis patients by MALDI-TOF-MSn. Analyst (2019) 144(6):2042–51. doi: 10.1039/C8AN02014K 30714583

[34] Trbojević Akmačić I Ventham NT Theodoratou E Vučković F Kennedy NA Krištić J. Inflammatory bowel disease associates with proinflammatory potential of the immunoglobulin G glycome. Inflamm Bowel Dis (2015) 21(6):1237–47. doi: 10.1097/MIB.0000000000000372 25895110 PMC4450892

[35] Clerc F Reiding KR Jansen BC Kammeijer GSM Bondt A Wuhrer M. Human plasma protein n-glycosylation. Glycoconj J (2016) 33(3):309–43. doi: 10.1007/s10719-015-9626-2 PMC4891372 26555091

[36] Varki A. Biological roles of glycans. Glycobiology (2017) 27(1):3–49. doi: 10.1093/glycob/cww086 27558841 PMC5884436

[37] Varki A. Sialic acids in human health and disease. Trends Mol Med (2008) 14(8):351–60. doi: 10.1016/j.molmed.2008.06.002 PMC2553044 18606570

[38] Cobb BA. The history of IgG glycosylation and where we are now. Glycobiology (2020) 30(4):202–13. doi: 10.1093/glycob/cwz065 PMC7109348 31504525

[39] Gudelj I Lauc G Pezer M. Immunoglobulin G glycosylation in aging and diseases. Cell Immunol (2018) 333:65–79. doi: 10.1016/j.cellimm.2018.07.009 30107893

[40] Mahan AE Tedesco J Dionne K Baruah K Cheng HD De Jager PL. A method for high-throughput, sensitive analysis of IgG fc and fab glycosylation by capillary electrophoresis. J Immunol Methods (2015) 417:34–44. doi: 10.1016/j.jim.2014.12.004 25523925 PMC5054724

[41] Baum LG Cobb BA. The direct and indirect effects of glycans on immune function. Glycobiology (2017) 27(7):619–24. doi: 10.1093/glycob/cwx036 28460052

[42] Hitsumoto Y Thompson SJ Zhang YW Rook GAW Elson CJ. RELATIONSHIP BETWEEN INTERLEUKIN 6, AGALACTOSYL IgG AND PRISTANE-INDUCED ARTHRITIS. Autoimmunity (1992) 11(4):247–54. doi: 10.3109/08916939209035162 1581469

[43] Grace PS Dolatshahi S Lu LL Cain A Palmieri F Petrone L. Antibody subclass and glycosylation shift following effective TB treatment. Front Immunol (2021) 12:679973. doi: 10.3389/fimmu.2021.679973 34290702 PMC8287567

[44] Bond A Alavi A Axford JS Bourke BE Bruckner FE Kerr MA. A detailed lectin analysis of IgG glycosylation, demonstrating disease specific changes in terminal galactose and n-acetylglucosamine. J Autoimmun (1997) 10(1):77–85. doi: 10.1006/jaut.1996.0104 9080302

[45] Gardinassi Luiz G Dotz V Hipgrave Ederveen A de Almeida Roque P Nery Costa Carlos H Costa Dorcas L. Clinical severity of visceral leishmaniasis is associated with changes in immunoglobulin G fc n-glycosylation. mBio (2014) 5(6):e01844–14. doi: 10.1128/mBio.01844-14 PMC4324239 25467439

[46] Ho C-H Chien R-N Cheng P-N Liu J-H Liu C-K Su C-S. Aberrant serum immunoglobulin G glycosylation in chronic hepatitis b is associated with histological liver damage and reversible by antiviral therapy. J Infect Dis (2015) 211(1):115–24. doi: 10.1093/infdis/jiu388 25015948

[47] Mehta AS Long RE Comunale MA Wang M Rodemich L Krakover J. Increased levels of galactose-deficient anti-gal immunoglobulin G in the sera of hepatitis c virus-infected individuals with fibrosis and cirrhosis. J Virol (2008) 82(3):1259–70. doi: 10.1128/JVI.01600-07 PMC2224448 18045939

[48] Scott DA Wang M Grauzam S Pippin S Black A Angel PM. GlycoFibroTyper: A novel method for the glycan analysis of IgG and the development of a biomarker signature of liver fibrosis. Front Immunol (2022) 13. doi: 10.3389/fimmu.2022.797460 PMC8858972 35197973

[49] Lin S Wang Y Wang X Yan B Lou W Di W. Serum immunoglobulin G n-glycome: a potential biomarker in endometrial cancer. Ann Trans Med (2020) 8(12):748–. doi: 10.21037/atm-20-3504 PMC7333120 32647673

[50] Vučković F Krištić J Gudelj I Teruel M Keser T Pezer M. Association of systemic lupus erythematosus with decreased immunosuppressive potential of the IgG glycome. Arthritis Rheumatol (2015) 67(11):2978–89. doi: 10.1002/art.39273 PMC4626261 26200652

[51] Vicente MM Alves I Gaifem J Rodrigues CS Fernandes Â Dias AM. Altered IgG glycosylation at COVID-19 diagnosis predicts disease severity. Eur J Immunol (2022) 52(6):946–957. doi: 10.1002/eji.202149491 35307819 PMC9087392

[52] Dekkers G Rispens T Vidarsson G. Novel concepts of altered immunoglobulin G galactosylation in autoimmune diseases. Front Immunol (2018) 9:553. doi: 10.3389/fimmu.2018.00553 29616041 PMC5867308

[53] Dekkers G Treffers L Plomp R Bentlage AEH de Boer M Koeleman CAM. Decoding the human immunoglobulin G-glycan repertoire reveals a spectrum of fc-receptor- and complement-Mediated-Effector activities. Front Immunol (2017) 8(877). doi: 10.3389/fimmu.2017.00877 PMC5539844 28824618

[54] Biermann MHC Griffante G Podolska MJ Boeltz S Stürmer J Muñoz LE. Sweet but dangerous – the role of immunoglobulin G glycosylation in autoimmunity and inflammation. Lupus (2016) 25(8):934–42. doi: 10.1177/0961203316640368 27252272

[55] Collin M Ehlers M. The carbohydrate switch between pathogenic and immunosuppressive antigen-specific antibodies. Exp Dermatol (2013) 22(8):511–4. doi: 10.1111/exd.12171 23808883

[56] De Jong SE Selman MHJ Adegnika AA Amoah AS Van Riet E Kruize YCM. IgG1 fc n-glycan galactosylation as a biomarker for immune activation. Sci Rep (2016) 6(1):28207. doi: 10.1038/srep28207 27306703 PMC4910062

[57] Tijardović M Marijančević D Bok D Kifer D Lauc G Gornik O. Intense physical exercise induces an anti-inflammatory change in IgG n-glycosylation profile. Front Physiol (2019) 10(1522). doi: 10.3389/fphys.2019.01522 PMC6933519 31920720

[58] Alter G Ottenhoff THM Joosten SA. Antibody glycosylation in inflammation, disease and vaccination. Semin Immunol (2018) 39:102–10. doi: 10.1016/j.smim.2018.05.003 PMC8731230 29903548

[59] Fokkink WJR Selman MHC Wuhrer M Jacobs BC. Immunoglobulin G fc n-glycosylation in Guillain-Barré syndrome treated with intravenous immunoglobulin. Clin Exp Immunol (2014) 178:105–7. doi: 10.1111/cei.12530 PMC4285510 25546781

[60] Blundell PA Le NPL Allen J Watanabe Y Pleass RJ. Engineering the fragment crystallizable (Fc) region of human IgG1 multimers and monomers to fine-tune interactions with sialic acid-dependent receptors. J Biol Chem (2017) 292(31):12994–3007. doi: 10.1074/jbc.M117.795047 PMC5546038 28620050

[61] Pleass RJ. The therapeutic potential of sialylated fc domains of human IgG. mAbs (2021) 13(1):1953220. doi: 10.1080/19420862.2021.1953220 34288809 PMC8296966

[62] Malphettes L Freyvert Y Chang J Liu P-Q Chan E Miller JC. Highly efficient deletion of FUT8 in CHO cell lines using zinc-finger nucleases yields cells that produce completely nonfucosylated antibodies. Biotechnol Bioeng (2010) 106(5):774–83. doi: 10.1002/bit.22751 20564614

[63] Beck A Reichert JM. Marketing approval of mogamulizumab. mAbs (2012) 4(4):419–25. doi: 10.4161/mabs.20996 PMC3499336 22699226

[64] Pereira NA Chan KF Lin PC Song Z. The “less-is-more” in therapeutic antibodies: Afucosylated anti-cancer antibodies with enhanced antibody-dependent cellular cytotoxicity. mAbs (2018) 10(5):693–711. doi: 10.1080/19420862.2018.1466767 29733746 PMC6150623

[65] Mimura Y Katoh T Saldova R O’Flaherty R Izumi T Mimura-Kimura Y. Glycosylation engineering of therapeutic IgG antibodies: challenges for the safety, functionality and efficacy. Protein Cell (2018) 9(1):47–62. doi: 10.1007/s13238-017-0433-3 28597152 PMC5777974

[66] Irvine EB Alter G. Understanding the role of antibody glycosylation through the lens of severe viral and bacterial diseases. Glycobiology (2020) 30(4):241–53. doi: 10.1093/glycob/cwaa018 PMC7109349 32103252

[67] Horn EJ Dempsey G Schotthoefer AM Prisco UL McArdle M Gervasi SS. The Lyme disease biobank: Characterization of 550 patient and control samples from the East coast and upper Midwest of the united states. J Clin Microbiol (2020) 58(6):e00032–20. doi: 10.1128/JCM.00032-20 PMC7269379 32102853

[68] Comunale MA Wang M Hafner J Krakover J Rodemich L Kopenhaver B. Identification and development of fucosylated glycoproteins as biomarkers of primary hepatocellular carcinoma. J Proteome Res (2009) 8(2):595–602. doi: 10.1021/pr800752c 19099421 PMC4427194

[69] Comunale MA Rodemich-Betesh L Hafner J Wang M Norton P Di Bisceglie AM. Linkage specific fucosylation of alpha-1-Antitrypsin in liver cirrhosis and cancer patients: Implications for a biomarker of hepatocellular carcinoma. PloS One (2010) 5(8):e12419. doi: 10.1371/journal.pone.0012419 20811639 PMC2928295

[70] Guile GR Rudd PM Wing DR Prime SB Dwek RA. A rapid high-resolution high-performance liquid chromatographic method for separating glycan mixtures and analyzing oligosaccharide profiles. Anal Biochem (1996) 240(2):210–26. doi: 10.1006/abio.1996.0351 8811911

[71] Powers TW Neely BA Shao Y Tang H Troyer DA Mehta AS. MALDI imaging mass spectrometry profiling of n-glycans in formalin-fixed paraffin embedded clinical tissue blocks and tissue microarrays. PloS One (2014) 9(9):e106255. doi: 10.1371/journal.pone.0106255 25184632 PMC4153616

[72] Black AP Liang H West CA Wang M Herrera HP Haab BB. A novel mass spectrometry platform for multiplexed n-glycoprotein biomarker discovery from patient biofluids by antibody panel based n-glycan imaging. Anal Chem (2019) 91(13):8429–35. doi: 10.1021/acs.analchem.9b01445 PMC7017646 31177770

[73] Black AP Angel PM Drake RR Mehta AS. Antibody panel based n -glycan imaging for n -glycoprotein biomarker discovery. Curr Protoc Protein Sci (2019) 98(1):e99. doi: 10.1002/cpps.99 31721442 PMC6859948

[74] Harvey DJ Merry AH Royle L P. Campbell M Dwek RA Rudd PM. Proposal for a standard system for drawing structural diagrams of n - and O -linked carbohydrates and related compounds. Proteomics (2009) 9(15):3796–801. doi: 10.1002/pmic.200900096 19670245

[75] Campbell MP Royle L Radcliffe CM Dwek RA Rudd PM. GlycoBase and autoGU: tools for HPLC-based glycan analysis. Bioinformatics (2008) 24(9):1214–6. doi: 10.1093/bioinformatics/btn090 18344517

[76] Yu X Wang Y Kristic J Dong J Chu X Ge S. Profiling IgG n-glycans as potential biomarker of chronological and biological ages: A community-based study in a han Chinese population. Medicine (Baltimore) (2016) 95(28):e4112. doi: 10.1097/MD.0000000000004112 27428197 PMC4956791

[77] Hastey CJ Elsner RA Barthold SW Baumgarth N. Delays and diversions mark the development of b cell responses toBorrelia burgdorferiInfection. J Immunol (2012) 188(11):5612–22. doi: 10.4049/jimmunol.1103735 PMC3358496 22547698

[78] Tunev SS Hastey CJ Hodzic E Feng S Barthold SW Baumgarth N. Lymphoadenopathy during Lyme borreliosis is caused by spirochete migration-induced specific b cell activation. PloS Pathog (2011) 7(5):e1002066. doi: 10.1371/journal.ppat.1002066 21637808 PMC3102705

[79] Comunale MA Wang M Anbarasan N Betesh L Karabudak A Moritz E. Total serum glycan analysis is superior to lectin-FLISA for the early detection of hepatocellular carcinoma. Proteomics Clin Appl (2013) 7(9-10):690–700. doi: 10.1002/prca.201200125 23857719 PMC4603546

[80] Shields RL Lai J Keck R O'Connell LY Hong K Meng YG. Lack of fucose on human IgG1 n-linked oligosaccharide improves binding to human FcγRIII and antibody-dependent cellular toxicity. J Biol Chem (2002) 277(30):26733–40. doi: 10.1074/jbc.M202069200 11986321

[81] Cao Y Song Z Guo Z Zhao X Gong Y Zhao K. Cytokines in the immune microenvironment change the glycosylation of IgG by regulating intracellular glycosyltransferases. Front Immunol (2022) 12. doi: 10.3389/fimmu.2021.724379 PMC8818798 35140700

[82] Bartsch YC Eschweiler S Leliavski A Lunding HB Wagt S Petry J. IgG fc sialylation is regulated during the germinal center reaction following immunization with different adjuvants. J Allergy Clin Immunol (2020) 146(3):652–66.e11. doi: 10.1016/j.jaci.2020.04.059 32445838

[83] Pfeifle R Rothe T Ipseiz N Scherer HU Culemann S Harre U. Regulation of autoantibody activity by the IL-23–TH17 axis determines the onset of autoimmune disease. Nat Immunol (2017) 18(1):104–13. doi: 10.1038/ni.3579 PMC5164937 27820809

[84] Keusch J Lydyard PM Delves PJ. The effect on IgG glycosylation of altering 1,4-galactosyltransferase-1 activity in b cells. Glycobiology (1998) 8(12):1207–13. doi: 10.1093/glycob/8.12.1215 9858643

[85] Dekkers G Plomp R Koeleman CAM Visser R Von Horsten HH Sandig V. Multi-level glyco-engineering techniques to generate IgG with defined fc-glycans. Sci Rep (2016) 6(1):36964. doi: 10.1038/srep36964 27872474 PMC5131652

[86] Omtvedt LA Royle L Husby G Sletten K Radcliffe CM Harvey DJ. Glycan analysis of monoclonal antibodies secreted in deposition disorders indicates that subsets of plasma cells differentially process IgG glycans. Arthritis Rheum (2006) 54(11):3433–40. doi: 10.1002/art.22171 17075835

[87] Elsner RA Hastey CJ Olsen KJ Baumgarth N. Suppression of long-lived humoral immunity following borrelia burgdorferi infection. PloS Pathog (2015) 11(7):e1004976. doi: 10.1371/journal.ppat.1004976 26136236 PMC4489802

[88] Anderson C Brissette CA. The brilliance of borrelia: Mechanisms of host immune evasion by Lyme disease-causing spirochetes. Pathogens (2021) 10(3):281. doi: 10.3390/pathogens10030281 33801255 PMC8001052

[89] Nothelfer K Sansonetti PJ Phalipon A. Pathogen manipulation of b cells: the best defence is a good offence. Nat Rev Microbiol (2015) 13(3):173–84. doi: 10.1038/nrmicro3415 25659322

[90] Ma Y Sturrock A Weis JJ. Intracellular localization of borrelia burgdorferi within human endothelial cells. Infect Immun (1991) 59(2):671–8. doi: 10.1128/iai.59.2.671-678.1991 PMC257809 1987083

[91] Petnicki-Ocwieja T Kern A. Mechanisms of borrelia burgdorferi internalization and intracellular innate immune signaling. Front Cell Infect Microbiol (2014) 4:175. doi: 10.3389/fcimb.2014.00175 25566512 PMC4266086

[92] Chiao JW Pavia C Riley M Altmann-Lasekan W Abolhassani M Liegner K. Antigens of Lyme disease of spirochaeteBorrelia burgdorferiinhibits antigen or mitogen-induced lymphocyte proliferation. FEMS Immunol Med Microbiol (1994) 8(2):151–5. doi: 10.1111/j.1574-695X.1994.tb00437.x 8173554

[93] Aberer E Koszik F Silberer M. Why is chronic Lyme borreliosis chronic? Clin Infect Dis (1997) 25(s1):S64–70. doi: 10.1086/516163 9233667

[94] Oefner CM Winkler A Hess C Lorenz AK Holecska V Huxdorf M. Tolerance induction with T cell–dependent protein antigens induces regulatory sialylated IgGs. J Allergy Clin Immunol (2012) 129(6):1647–55.e13. doi: 10.1016/j.jaci.2012.02.037 22502800

[95] Elsner RA Hastey CJ Baumgarth N. CD4+ T cells promote antibody production but not sustained affinity maturation during borrelia burgdorferi infection. Infect Immun (2015) 83(1):48–56. doi: 10.1128/IAI.02471-14 25312948 PMC4288900

[96] Chatterjee S Kawahara R Tjondro HC Shaw DR Nenke MA Torpy DJ. Serum n-glycomics stratifies bacteremic patients infected with different pathogens. J Clin Med (2021) 10(3):516. doi: 10.3390/jcm10030516 33535571 PMC7867038

[97] Ramirez J Guarner F Bustos Fernandez L Maruy A Sdepanian VL Cohen H. Antibiotics as major disruptors of gut microbiota. Front Cell Infect Microbiol (2020) 10. doi: 10.3389/fcimb.2020.572912 PMC7732679 33330122

