# Supplementary material for: Acute Lyme disease IgG N-linked glycans contrast the canonical inflammatory signature
Source: Front Immunol. 2022 Aug 5;13:949118. doi: 10.3389/fimmu.2022.949118 (PMC9389449; doi:10.3389/fimmu.2022.949118)
Supplement: Supplementary file 1 [file Presentation_1.pptx]

## Slide 1
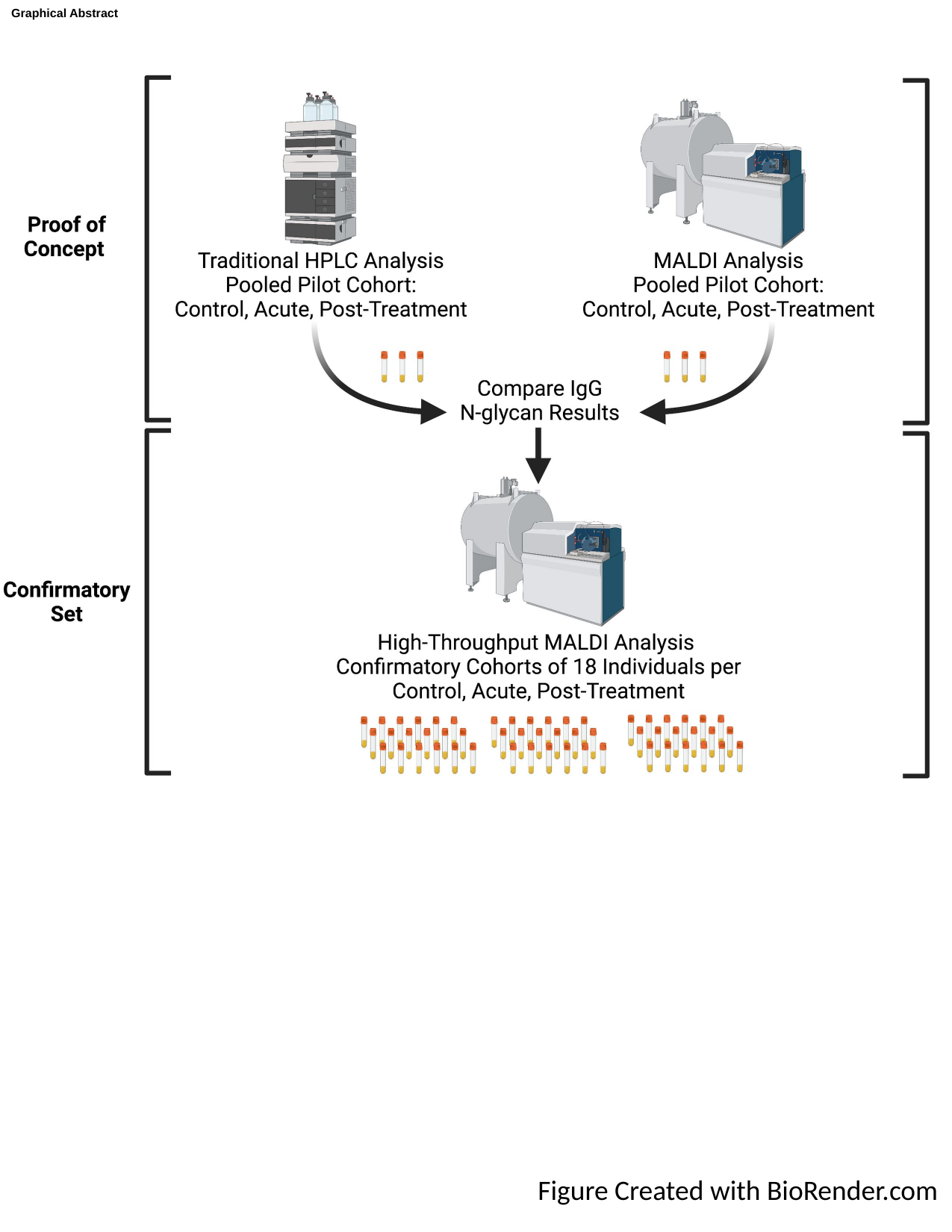

Graphical Abstract
Figure Created with BioRender.com

## Slide 2
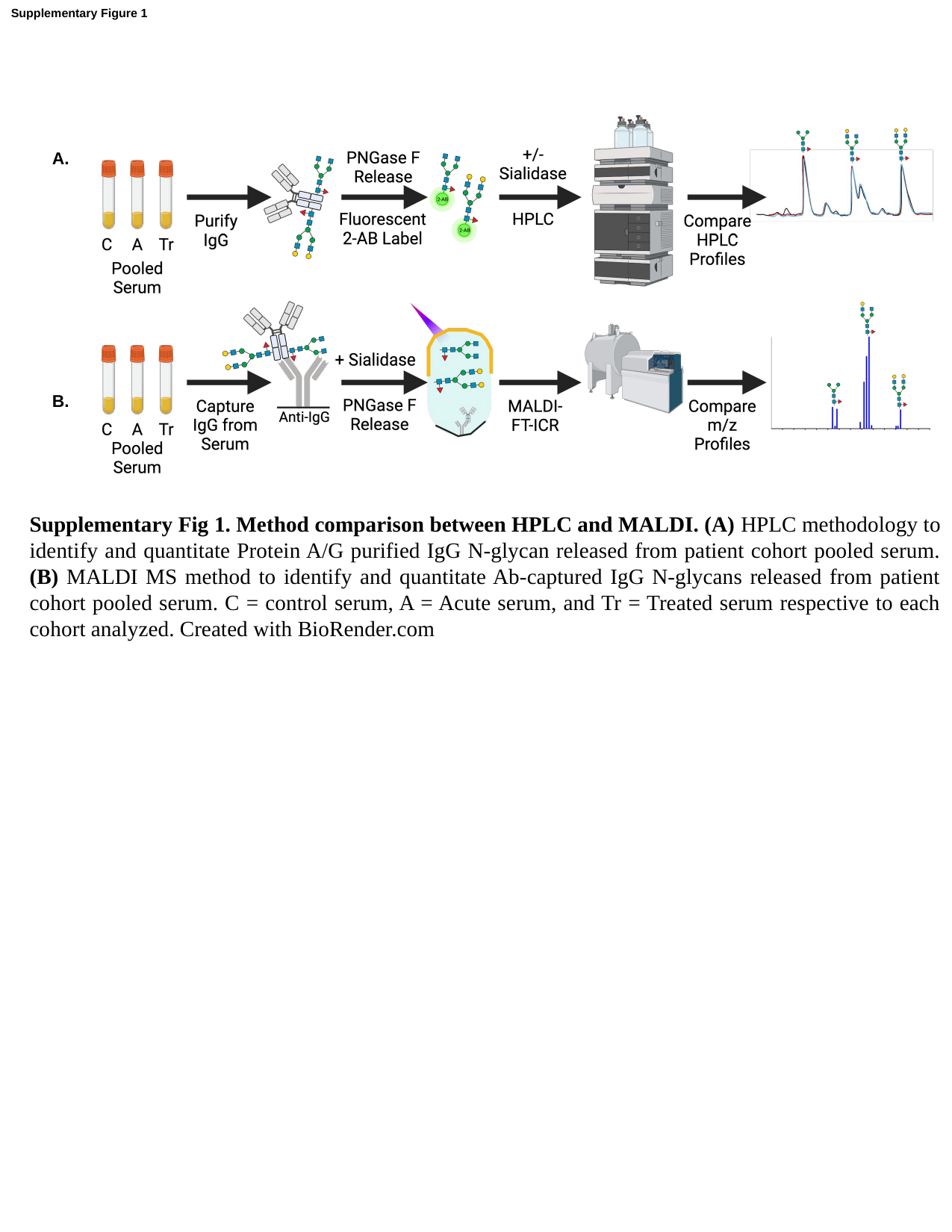

Supplementary Figure 1
A.
B.
Supplementary Fig 1. Method comparison between HPLC and MALDI. (A) HPLC methodology to identify and quantitate Protein A/G purified IgG N-glycan released from patient cohort pooled serum. (B) MALDI MS method to identify and quantitate Ab-captured IgG N-glycans released from patient cohort pooled serum. C = control serum, A = Acute serum, and Tr = Treated serum respective to each cohort analyzed. Created with BioRender.com

## Slide 3
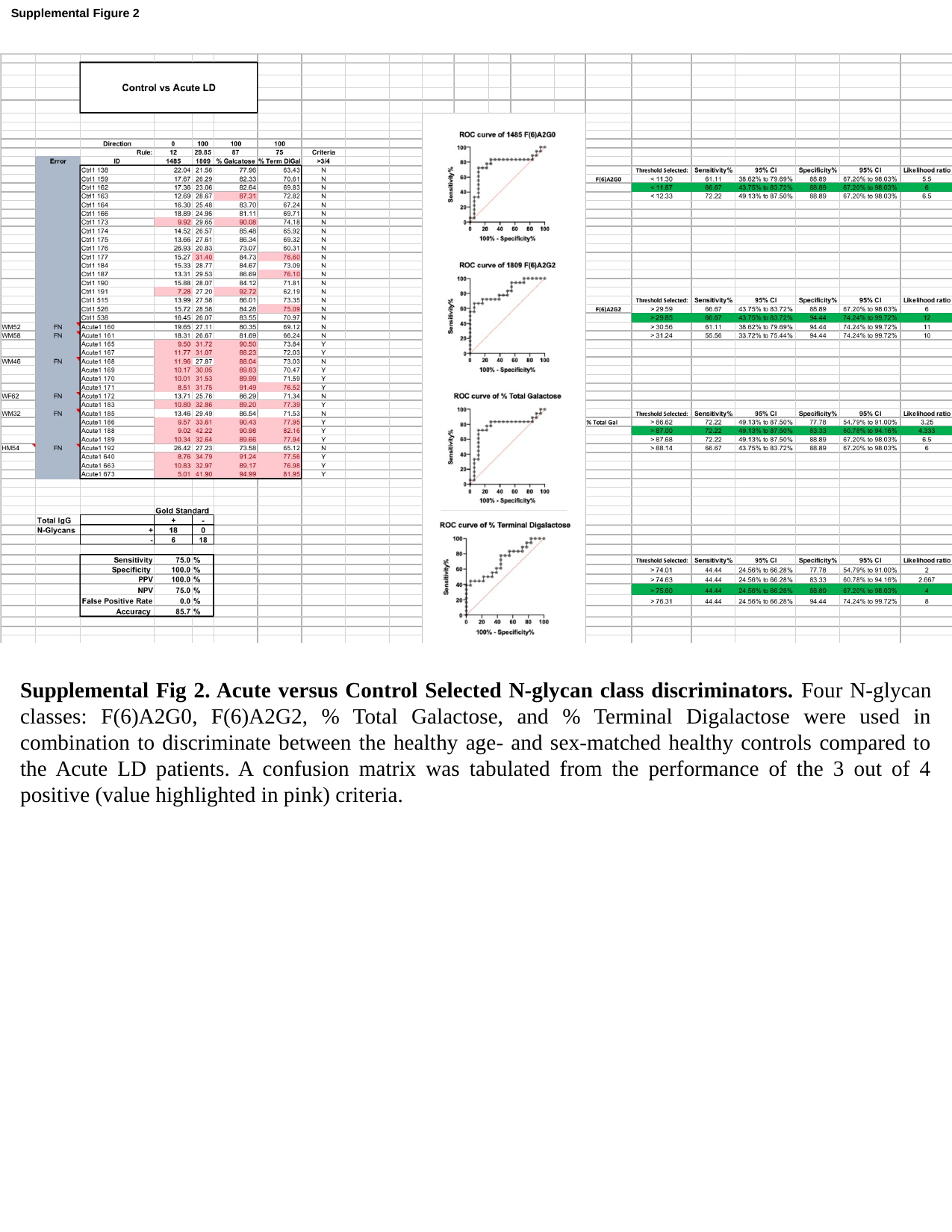

Supplemental Figure 2
Supplemental Fig 2. Acute versus Control Selected N-glycan class discriminators. Four N-glycan classes: F(6)A2G0, F(6)A2G2, % Total Galactose, and % Terminal Digalactose were used in combination to discriminate between the healthy age- and sex-matched healthy controls compared to the Acute LD patients. A confusion matrix was tabulated from the performance of the 3 out of 4 positive (value highlighted in pink) criteria.

## Slide 4
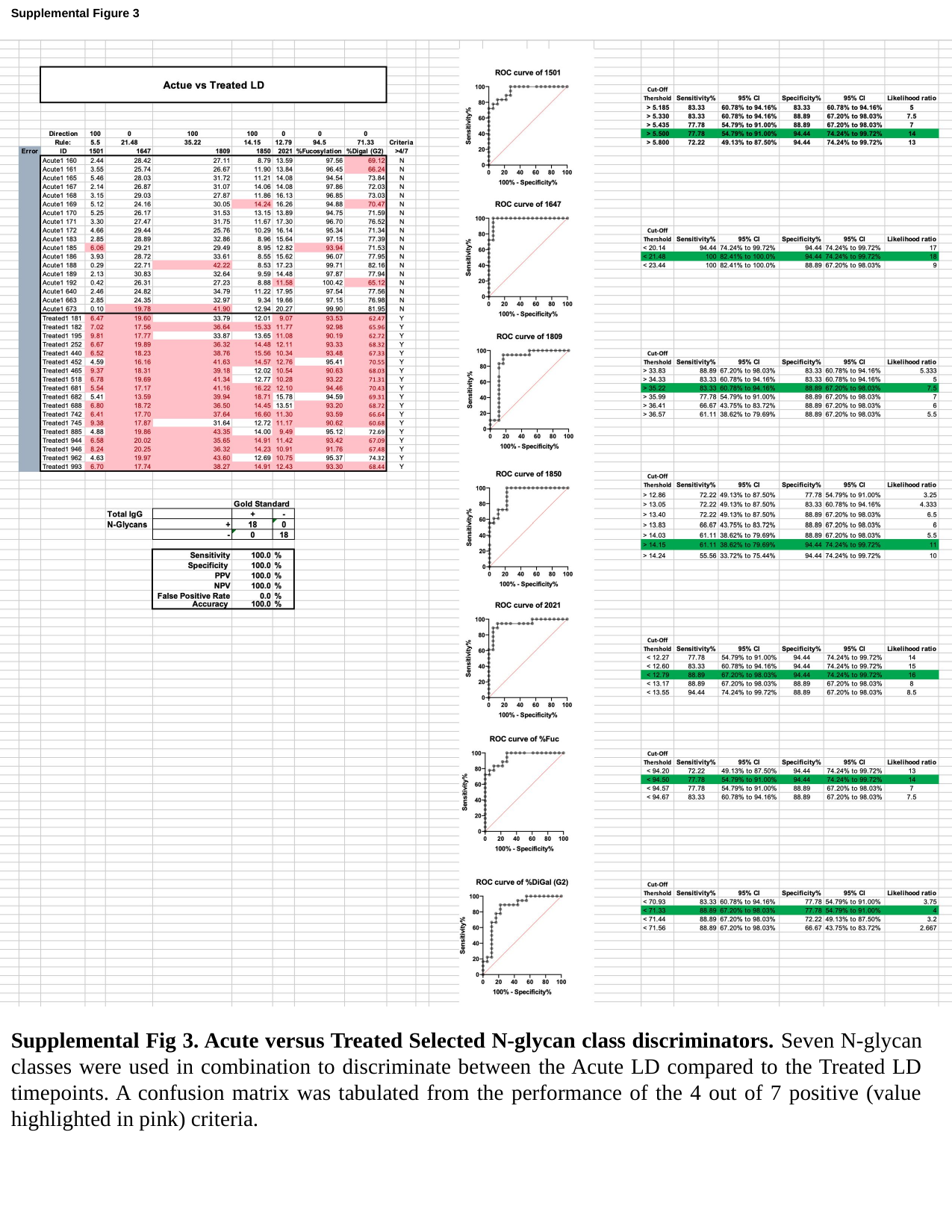

Supplemental Figure 3
Supplemental Fig 3. Acute versus Treated Selected N-glycan class discriminators. Seven N-glycan classes were used in combination to discriminate between the Acute LD compared to the Treated LD timepoints. A confusion matrix was tabulated from the performance of the 4 out of 7 positive (value highlighted in pink) criteria.

## Slide 5
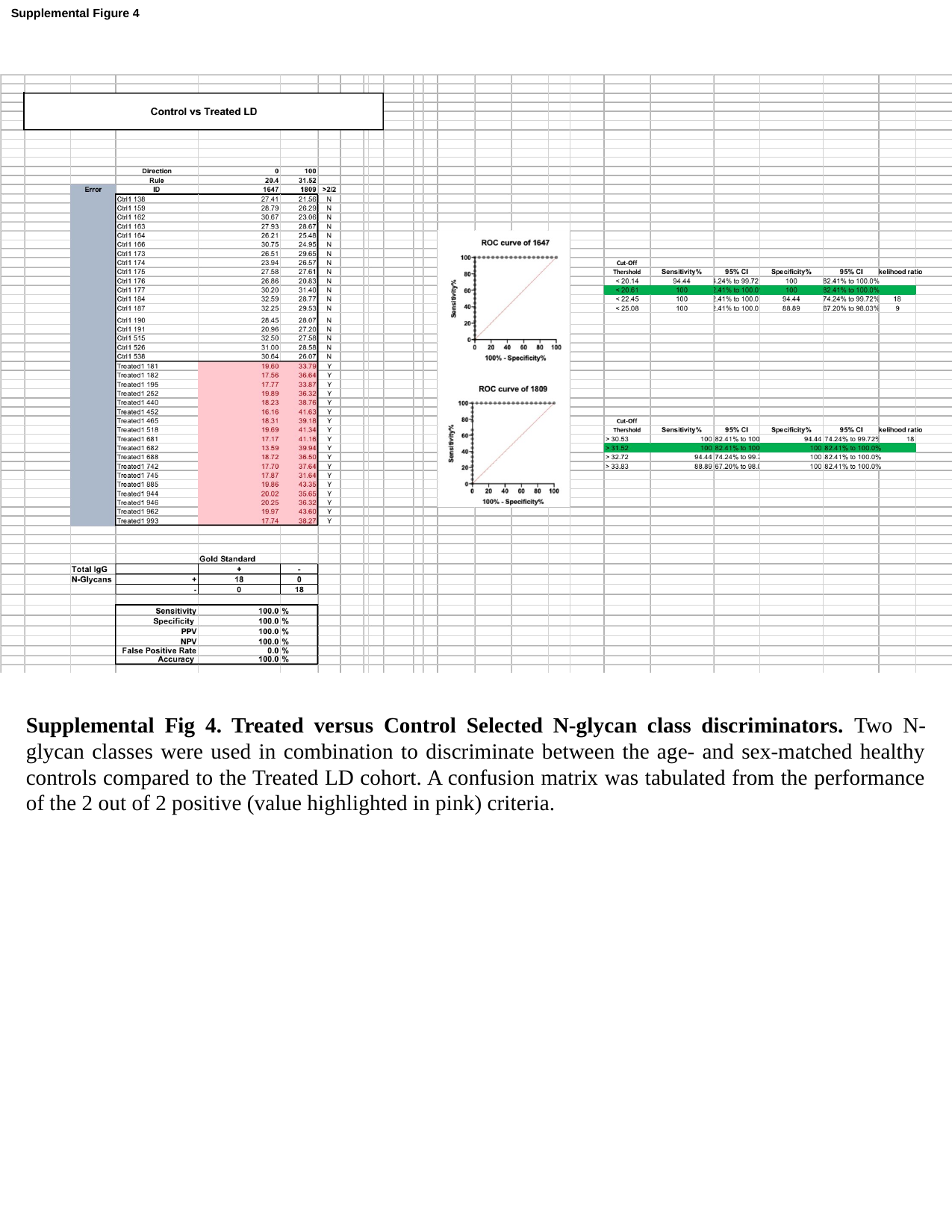

Supplemental Figure 4
Supplemental Fig 4. Treated versus Control Selected N-glycan class discriminators. Two N-glycan classes were used in combination to discriminate between the age- and sex-matched healthy controls compared to the Treated LD cohort. A confusion matrix was tabulated from the performance of the 2 out of 2 positive (value highlighted in pink) criteria.

## Slide 6
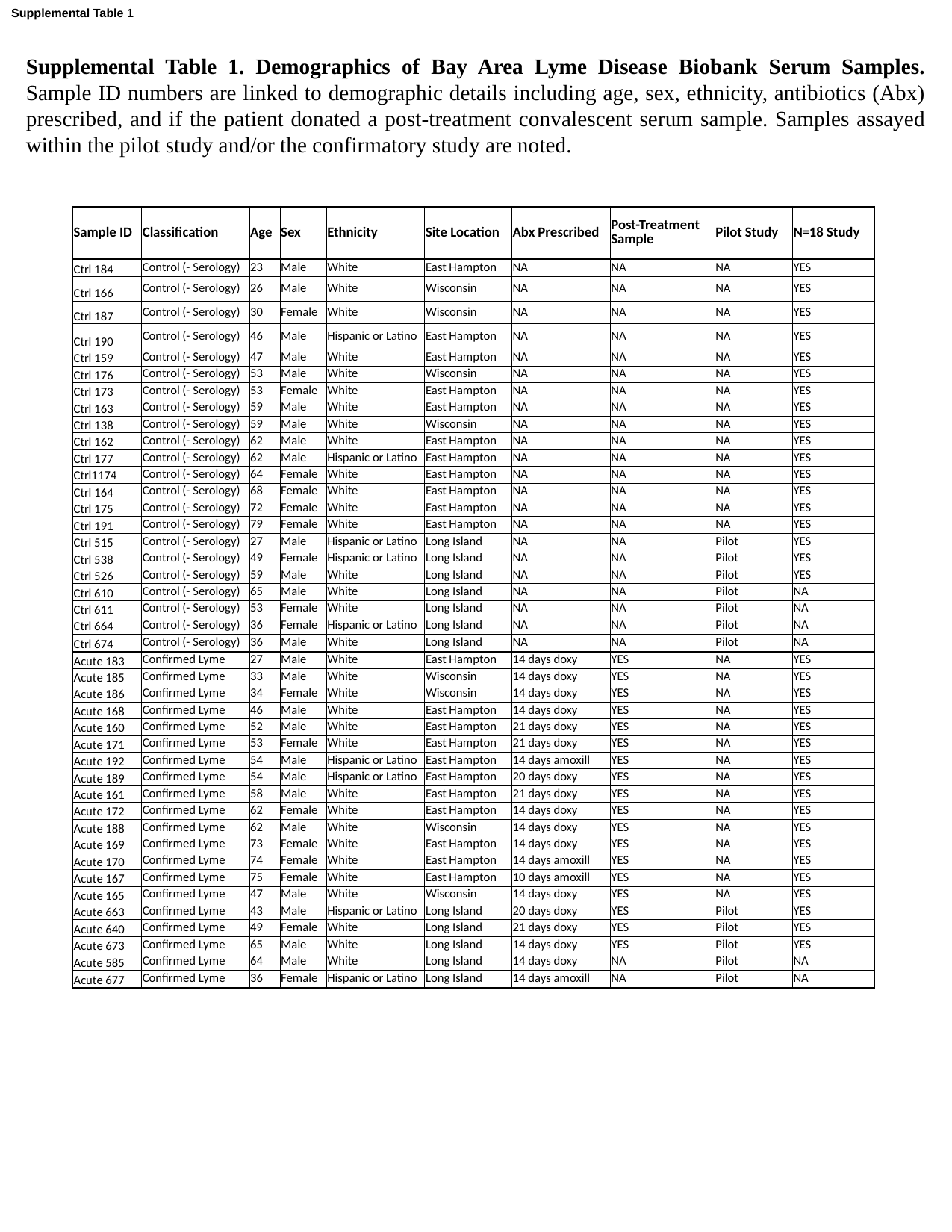

Supplemental Table 1
Supplemental Table 1. Demographics of Bay Area Lyme Disease Biobank Serum Samples. Sample ID numbers are linked to demographic details including age, sex, ethnicity, antibiotics (Abx) prescribed, and if the patient donated a post-treatment convalescent serum sample. Samples assayed within the pilot study and/or the confirmatory study are noted.
| Sample ID | Classification | Age | Sex | Ethnicity | Site Location | Abx Prescribed | Post-Treatment Sample | Pilot Study | N=18 Study |
| --- | --- | --- | --- | --- | --- | --- | --- | --- | --- |
| Ctrl 184 | Control (- Serology) | 23 | Male | White | East Hampton | NA | NA | NA | YES |
| Ctrl 166 | Control (- Serology) | 26 | Male | White | Wisconsin | NA | NA | NA | YES |
| Ctrl 187 | Control (- Serology) | 30 | Female | White | Wisconsin | NA | NA | NA | YES |
| Ctrl 190 | Control (- Serology) | 46 | Male | Hispanic or Latino | East Hampton | NA | NA | NA | YES |
| Ctrl 159 | Control (- Serology) | 47 | Male | White | East Hampton | NA | NA | NA | YES |
| Ctrl 176 | Control (- Serology) | 53 | Male | White | Wisconsin | NA | NA | NA | YES |
| Ctrl 173 | Control (- Serology) | 53 | Female | White | East Hampton | NA | NA | NA | YES |
| Ctrl 163 | Control (- Serology) | 59 | Male | White | East Hampton | NA | NA | NA | YES |
| Ctrl 138 | Control (- Serology) | 59 | Male | White | Wisconsin | NA | NA | NA | YES |
| Ctrl 162 | Control (- Serology) | 62 | Male | White | East Hampton | NA | NA | NA | YES |
| Ctrl 177 | Control (- Serology) | 62 | Male | Hispanic or Latino | East Hampton | NA | NA | NA | YES |
| Ctrl1174 | Control (- Serology) | 64 | Female | White | East Hampton | NA | NA | NA | YES |
| Ctrl 164 | Control (- Serology) | 68 | Female | White | East Hampton | NA | NA | NA | YES |
| Ctrl 175 | Control (- Serology) | 72 | Female | White | East Hampton | NA | NA | NA | YES |
| Ctrl 191 | Control (- Serology) | 79 | Female | White | East Hampton | NA | NA | NA | YES |
| Ctrl 515 | Control (- Serology) | 27 | Male | Hispanic or Latino | Long Island | NA | NA | Pilot | YES |
| Ctrl 538 | Control (- Serology) | 49 | Female | Hispanic or Latino | Long Island | NA | NA | Pilot | YES |
| Ctrl 526 | Control (- Serology) | 59 | Male | White | Long Island | NA | NA | Pilot | YES |
| Ctrl 610 | Control (- Serology) | 65 | Male | White | Long Island | NA | NA | Pilot | NA |
| Ctrl 611 | Control (- Serology) | 53 | Female | White | Long Island | NA | NA | Pilot | NA |
| Ctrl 664 | Control (- Serology) | 36 | Female | Hispanic or Latino | Long Island | NA | NA | Pilot | NA |
| Ctrl 674 | Control (- Serology) | 36 | Male | White | Long Island | NA | NA | Pilot | NA |
| Acute 183 | Confirmed Lyme | 27 | Male | White | East Hampton | 14 days doxy | YES | NA | YES |
| Acute 185 | Confirmed Lyme | 33 | Male | White | Wisconsin | 14 days doxy | YES | NA | YES |
| Acute 186 | Confirmed Lyme | 34 | Female | White | Wisconsin | 14 days doxy | YES | NA | YES |
| Acute 168 | Confirmed Lyme | 46 | Male | White | East Hampton | 14 days doxy | YES | NA | YES |
| Acute 160 | Confirmed Lyme | 52 | Male | White | East Hampton | 21 days doxy | YES | NA | YES |
| Acute 171 | Confirmed Lyme | 53 | Female | White | East Hampton | 21 days doxy | YES | NA | YES |
| Acute 192 | Confirmed Lyme | 54 | Male | Hispanic or Latino | East Hampton | 14 days amoxill | YES | NA | YES |
| Acute 189 | Confirmed Lyme | 54 | Male | Hispanic or Latino | East Hampton | 20 days doxy | YES | NA | YES |
| Acute 161 | Confirmed Lyme | 58 | Male | White | East Hampton | 21 days doxy | YES | NA | YES |
| Acute 172 | Confirmed Lyme | 62 | Female | White | East Hampton | 14 days doxy | YES | NA | YES |
| Acute 188 | Confirmed Lyme | 62 | Male | White | Wisconsin | 14 days doxy | YES | NA | YES |
| Acute 169 | Confirmed Lyme | 73 | Female | White | East Hampton | 14 days doxy | YES | NA | YES |
| Acute 170 | Confirmed Lyme | 74 | Female | White | East Hampton | 14 days amoxill | YES | NA | YES |
| Acute 167 | Confirmed Lyme | 75 | Female | White | East Hampton | 10 days amoxill | YES | NA | YES |
| Acute 165 | Confirmed Lyme | 47 | Male | White | Wisconsin | 14 days doxy | YES | NA | YES |
| Acute 663 | Confirmed Lyme | 43 | Male | Hispanic or Latino | Long Island | 20 days doxy | YES | Pilot | YES |
| Acute 640 | Confirmed Lyme | 49 | Female | White | Long Island | 21 days doxy | YES | Pilot | YES |
| Acute 673 | Confirmed Lyme | 65 | Male | White | Long Island | 14 days doxy | YES | Pilot | YES |
| Acute 585 | Confirmed Lyme | 64 | Male | White | Long Island | 14 days doxy | NA | Pilot | NA |
| Acute 677 | Confirmed Lyme | 36 | Female | Hispanic or Latino | Long Island | 14 days amoxill | NA | Pilot | NA |
